# Extended Follow-Up and Analysis of Non-secretory IgG-Type Multiple Myeloma in a Patient With Fibromyalgia: A Case Report

**DOI:** 10.7759/cureus.63321

**Published:** 2024-06-27

**Authors:** Ryuichi Ohta, Yumi Naito, Chiaki Sano

**Affiliations:** 1 Community Care, Unnan City Hospital, Unnan, JPN; 2 Community Medicine Management, Shimane University Faculty of Medicine, Izumo, JPN

**Keywords:** general medicine, family medicine, rural, diagnostic imaging, pain management, anemia, c-reactive protein, multiple myeloma, fibromyalgia

## Abstract

Fibromyalgia (FM) presents a diagnostic challenge due to its complex symptoms and lack of definitive tests. This study discusses a 54-year-old female initially diagnosed with FM, characterized by widespread pain, fatigue, and tender points. Despite treatment, she developed elevated C-reactive protein (CRP) and anemia after two years, leading to further investigations. These tests revealed non-secretory multiple myeloma, underscoring the importance of vigilant monitoring in FM patients. This case highlights the need for regular CRP measurements and thorough follow-up to detect underlying conditions. Early detection and appropriate intervention are crucial in managing FM and improving patient outcomes.

## Introduction

Fibromyalgia (FM) is a challenging condition for general physicians to diagnose and treat, characterized by widespread musculoskeletal pain, fatigue, and localized tenderness [1]. The complexity of FM symptoms coupled with the absence of definitive diagnostic tests complicates management. Triggers for FM include stress, trauma, infections, and genetic factors, leading to highly individualized symptom presentations [2].

The diagnosis of FM hinges on the lack of inflammatory markers, differentiating it from other rheumatic and autoimmune diseases [3]. Regular monitoring is essential for managing FM, as it allows for careful symptom assessment and prompt response to changes in the patient's condition [4]. The presence of inflammatory markers like C-reactive protein (CRP) in FM patients can signal the development of other health issues [5].

This study discusses a middle-aged female FM patient who developed elevated CRP and anemia and was eventually diagnosed with non-secretory multiple myeloma during a two-year follow-up. Her case underscores the importance of vigilance in monitoring FM patients for other underlying conditions. Elevated CRP prompted further diagnostic evaluations, leading to the discovery of this rare cancer [6]. This report highlights the critical role of regular, thorough monitoring in FM management to improve patient outcomes.

## Case presentation

A 54-year-old female came to a rural community hospital with a chief complaint of systemic joint pains and fatigue for two months. Three months before the first visit to the hospital, the patient had insomnia and fatigue with gradual onset. Two months before the first visit to the hospital, systemic joint pains with gradual onset developed, and the pain was severe at most in the morning but persistent in the daytime. Her activity in daily life was impaired so she came to the hospital with the help of her husband. At the initial presentation, she had no signs of infections, fever, night sweats, or body weight loss. She has a depressed mood but did not have any ideation of suicide. She did not have any past medical history. She did not take any medication at the first visit.

The vital signs at the visit were as follows: blood pressure 110/65 mmHg, pulse rate 69 beats/min, body temperature 36.9°C, respiratory rate 15 breaths/min, and oxygen saturation 97% on room air. The patient was alert to time, place, and person. Physical examination showed multiple tenderness on bilateral peri-wrist, elbow, shoulder, and knee lesions. In addition, there was tenderness on the posterior parts of the vertebras from the neck to the sacroiliac joints. There were no findings of arthritis. No other abnormal neurological findings were noted; no apparent chest or abdomen abnormalities or skin eruptions.

The laboratory tests showed no elevation of inflammatory markers such as C-reactive protein (CRP) and autoimmune antibodies such as antinuclear antibodies, rheumatoid factors, and anti-citrullinated protein antibodies. The hands, shoulders, knees, and spine X-rays showed no abrasions or osteophyte formations. Magnetic resonance imaging (MRI) of the same lesions revealed no signs of inflammation.

Based on the clinical findings, she was diagnosed with fibromyalgia. The patient was treated with acetaminophen, pregabalin, and duloxetine. By increasing the dose of the medication, the symptoms were alleviated and followed at the outpatient department. Her symptoms had been controlled with the medicines of acetaminophen of 1000 mg, pregabalin of 300 mg, and duloxetine of 20 mg daily.

Two years later, her pain gradually worsened, especially in the spine and pelvis. Her laboratory tests showed mildly increased CRP (0.45 mg/dL) and mild anemia (11.2 g/dL). Two months after the exacerbation, her white blood cells and platelet counts decreased gradually, and inflammatory conditions persisted (Table 1).

**Table 1:** Laboratory data of the patient. eGFR: estimated glomerular filtration rate; CK: creatine kinase; CRP: C-reactive protein; TSH: thyroid-stimulating hormone; Ig: immunoglobulin; HCV: hepatitis C virus; SARS-CoV-2: severe acute respiratory syndrome coronavirus 2; HIV: human immunodeficiency virus; HBs: hepatitis B surface antigen; HBc: hepatitis B core antigen; C3: complement component 3; C4: complement component 4; MPO-ANCA: myeloperoxidase anti-neutrophil cytoplasmic antibody; CCP: cyclic citrullinated peptide

Parameter	Level	Reference
White blood cells	3.00×10^3^/μL	3.5-9.1×10^3^/μL
Neutrophils	46.1%	44.0-72.0%
Lymphocytes	38.7%	18.0-59.0%
Monocytes	14.2%	0.0-12.0%
Eosinophils	0.5%	0.0-10.0%
Basophils	0.5%	0.0-3.0%
Red blood cells	2.98×10^6^/μL	3.76-5.50×10^6^/μL
Hemoglobin	9.6 g/dL	11.3-15.2 g/dL
Hematocrit	27.6%	33.4-44.9%
Mean corpuscular volume	93.6 fL	79.0-100.0 fL
Platelets	11.6×10^4^/μL	13.0-36.9×10^4^/μL
Erythrocyte sedimentation rate	45 mm/h	2-10 mm/h
Total protein	7.4 g/dL	6.5-8.3 g/dL
Albumin	4.4 g/dL	3.8-5.3 g/dL
Total bilirubin	0.7 mg/dL	0.2-1.2 mg/dL
Aspartate aminotransferase	30 IU/L	8-38 IU/L
Alanine aminotransferase	23 IU/L	4-43 IU/L
Alkaline phosphatase	69 IU/L	106-322 U/L
γ-Glutamyl transpeptidase	48 IU/L	<48 IU/L
Lactate dehydrogenase	269 U/L	121-245 U/L
Blood urea nitrogen	11.8 mg/dL	8-20 mg/dL
Creatinine	0.62 mg/dL	0.40-1.10 mg/dL
eGFR	76.1 mL/min/L	>60.0 mL/min/L
Serum Na	140 mEq/L	135-150 mEq/L
Serum K	3.8 mEq/L	3.5-5.3 mEq/L
Serum Cl	105 mEq/L	98-110 mEq/L
Serum Ca	9.5 mg/dL	8.8-10.2 mg/dL
Serum P	3.6 mg/dL	2.7-4.6 mg/dL
Serum Mg	2.1 mg/dL	1.8-2.3 mg/dL
Ferritin	363.0 ng/dL	14.4-303.7 ng/mL
CK	123 U/L	56-244 U/L
CRP	0.56 mg/dL	<0.30 mg/dL
TSH	1.88 μIU/mL	0.35-4.94 μIU/mL
Free T4	3.2 ng/dL	0.70-1.48 ng/dL
IgG	1686 mg/dL	870-1700 mg/dL
IgM	28 mg/dL	35-220 mg/dL
IgA	66 mg/dL	110-410 mg/dL
IgE	31 mg/dL	<173 mg/dL
HBs antigen	0.0 IU/mL	<0.05 IU/mL
HBs antibody	0.00 mIU/mL	<10.0 mIU/mL
HBc antibody	0.00	<1 S/CO
HCV antibody	0.00	<1 S/CO
Syphilis treponema antibody	0.00	S/CO
SARS-CoV-2 antigen	Negative	Negative
Anti-nuclear antibody	40	<40
C3	126 mg/dL	86-164 mg/dL
C4	27 mg/dL	17-45 mg/dL
MPO-ANCA	<1.0 U/mL	<3.5 U/mL
Anti-CCP antibody	<0.6 U/mL	<5 U/mL
Urine test		
Leukocyte	Negative	Negative
Nitrite	Negative	Negative
Protein	Negative	Negative
Glucose	Negative	Negative
Bilirubin	Negative	Negative
Blood	Negative	Negative
pH	6.5	-
Specific gravity	1.013	-

**Figure 1:**
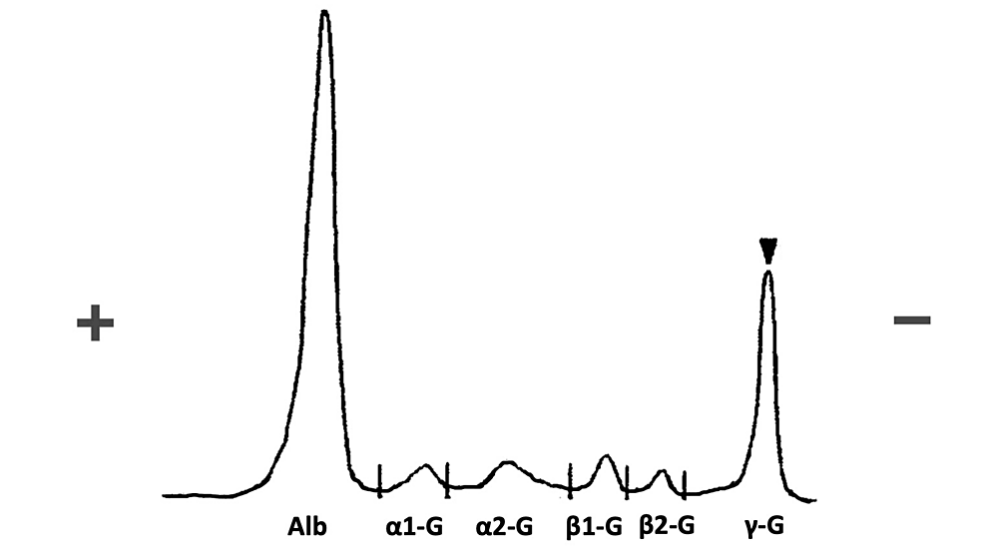
The result of the immunoelectrophoresis showing the presence of M protein (arrowhead).

Suspecting hematological diseases, peripheral blood smears, immunoglobulin levels, and the presence of M protein were investigated. The results show immunoglobulin G was mildly increased, and immunoglobulin A and M were decreased with the positive result of M protein (Figure 1).

**Figure 2:**
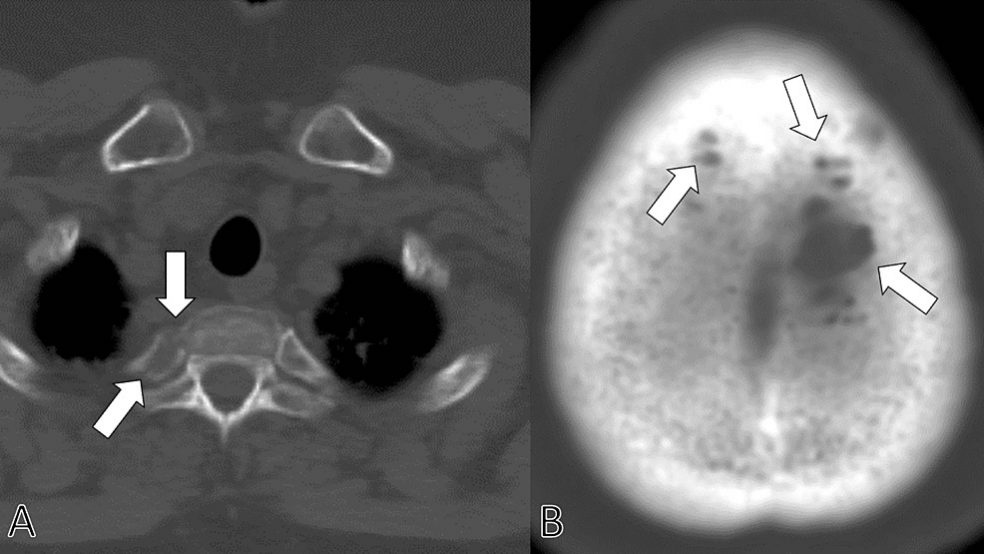
Systemic computed tomography clarifying multiple bone lytic lesions on the ribs (A) and skull (B) (arrows).

Suspecting multiple myeloma, the patient was consulted by the hematology department. Systemic computed tomography was performed in the hematology department, clarifying multiple bone lytic lesions on the vertebra and skull (Figures 2A, 2B).

She was suspected of non-secretory multiple myeloma and transferred to a tertiary care hospital. At the tertiary hospital, she was admitted and treated with daratumumab, bortezomib, lenalidomide, and dexamethasone, which alleviated her symptoms and followed in the hospital's outpatient department.

## Discussion

This case report highlights the intricate and multifaceted nature of diagnosing and managing FM, particularly in the context of comorbid conditions that may arise [7]. The patient's initial presentation, characterized by widespread musculoskeletal pain, fatigue, and specific tender points, aligns with the diagnostic criteria for FM. FM poses significant challenges for general physicians due to its symptom overlap with various other disorders and the absence of definitive diagnostic tests [8].

In general medicine, differential diagnosis should be emphasized and decisive in patients with multiple symptoms. The complexity of FM is underscored by its varied symptomatology and potential triggers, including stress, trauma, infections, and genetic predispositions [9]. This variability necessitates a comprehensive differential diagnosis to exclude other conditions, particularly those with similar clinical features but different underlying pathophysiology [10]. In this case, the lack of inflammatory markers and absence of clinical signs of inflammation initially supported the FM diagnosis, consistent with existing literature emphasizing FM's non-inflammatory nature. However, the eventual diagnosis of multiple myeloma (MM) could be detected through continual monitoring of inflammatory conditions. Thus, general physicians should not be decisive and follow-up on patients’ multiple symptoms to detect alarming symptoms [9].

Monitoring and management of FM should include the inflammatory conditions. As this case illustrates, regular monitoring of FM patients regarding CRP is crucial [11]. The patient’s gradual exacerbation of pain, particularly in the spine and pelvis, coupled with new-onset anemia and elevated CRP levels, warranted further investigation beyond the scope of FM. The diagnosis of FM is performed, ruling out various critical and inflammatory diseases [3]. This vigilant approach is vital in managing FM as it allows for the early detection of atypical symptoms that may indicate the presence of other severe conditions, including autoimmune and malignant diseases.

The role of inflammatory markers should be emphasized in managing patients with multiple symptoms such as fatigue, mild fever, and pain. The elevation of CRP in this patient was a key indicator prompting further diagnostic workup. CRP is a well-established inflammation marker and can signal additional health issues in FM patients [12]. The persistent elevation of CRP and concurrent development of anemia raised suspicions of a hematologic disorder, leading to the diagnosis of non-secretary multiple myeloma [13]. Although inflammatory markers can mislead physicians to do excessive testing on patients without critical diseases. However, deterioration of symptoms with positive CRP results should be investigated vigilantly. This case underscores the necessity of considering inflammatory markers in the follow-up of FM patients, even though FM itself is not associated with inflammatory changes [14].

The diagnosis of non-secretary multiple myeloma in this patient underscores the importance of maintaining a high index of suspicion for other underlying conditions in FM patients presenting with atypical symptoms. Non-secretory multiple myeloma is a rare subtype of multiple myeloma that does not produce detectable levels of myeloma proteins in the blood, making it challenging to diagnose [15]. Multiple bone lytic lesions on computed tomography further supported this diagnosis, highlighting the importance of comprehensive diagnostic imaging in such cases [16].

## Conclusions

This study reinforces the critical role of thorough and continuous monitoring in the management of FM. It illustrates how changes in clinical presentation, particularly the emergence of inflammatory markers and anemia, should prompt a re-evaluation of the initial diagnosis, leading to non-secretary multiple myeloma. General physicians must be vigilant in assessing FM patients for any signs of comorbid conditions to ensure timely and appropriate intervention. Future research should focus on developing more precise diagnostic tools and treatment strategies to manage the complex interplay of symptoms in FM and associated conditions.
